# Clinical Presentation of Ulcerative Colitis in Pakistani Adults

**DOI:** 10.5005/jp-journals-10018-1151

**Published:** 2016-07-09

**Authors:** Mustafa Qureshi, Zaigham Abbas

**Affiliations:** 1 Department of Medicine, Orthopedic and Medical Institute, Karachi, Pakistan

**Keywords:** Clinical presentation, Pakistan, Ulcerative colitis.

## Abstract

**Objective:**

The aim of this study was to determine the clinical presentation and severity of ulcerative colitis (UC) in Pakistani adult patients.

**Materials and methods:**

An observational study. Data were obtained by reviewing the medical records of patients who visited a gastroenterology clinic between 2008 and 2012.

**Results:**

There were 54 patients diagnosed as UC. The male to female ratio was 1:1. Mean age at diagnosis of UC was 38.7 ± 11.8 years (median 36.5, range 18-64). The predominant presenting symptoms were mucus diarrhea in 49 (90.7%), gross blood in stools in 42 (77.8%), abdominal pain or cramps in 40 (74.1%) and weight loss in 15 (27.7%). Left-sided colitis was present in 23 (42.6%), pancolitis in 15 (27.8%), extensive colitis in 11 (20.4%), and proctitis in five (9.2%). The severity of UC as judged by the Mayo scoring system showed that 68.5% were suffering from moderate to severe disease while 31.5% had mild disease. The extra-intestinal manifestation were found only in seven patients; arthritis in five patients and anterior uveitis in two patients. The arthritis was unilateral and the sites were knee joint in three patients and sacroiliac joint in two patients.

**Conclusion:**

Ulcerative colitis presents in our adult patients may present at any age with no gender preponderance. The disease severity is moderate to severe in the majority of patients and more than half of them have left-sided colitis or pancolitis at the time of presentation. Extraintestinal manifestations were not common.

**How to cite this article:**

Qureshi M, Abbas Z. Clinical Presentation of Ulcerative Colitis in Pakistani Adults. Euroasian J Hepato-Gastroenterol 2015;5(2):127-130.

## INTRODUCTION

The idiopathic inflammatory bowel disease (IBD) has been primarily characterized as a disease of industrialized nations, with increased prevalence in the developed world. Although developing regions have traditionally reported lower prevalence of IBD, the incidence of IBD is rising in many of these nations (e.g. India and China) as they have become industrialized.^1^ The etiology of IBD is unknown and interplay of genetic and environmental factors has been implicated.^2^ A study done by Montgomery et al reported that young Asians with ethnic origin in Indian Subcontinent who were born in Britain were at a significantly higher risk of developing IBD than the indigenous European population and suggested the role of environmental factors in unveiling the genetic predisposition to disease.^3^ A prevailing hypothesis is that the intestinal inflammation represents an inappropriate immune response to normal luminal bacteria in genetically susceptible individuals.^4^ Thus, the role of dietary habits alone on the development of IBD, which has been extensively investigated in case-control retrospective studies, is subject to different biases.^5^ Not enough data is available in this regard in local literature. The aim of this study was to determine the clinical presentation and severity of UC in Pakistani adult patients visiting a busy GI Clinic.

## MATERIALS AND METHODS

Retrospective data were obtained from the vigilant review of all adult patients with UC who attended a busy gastroenterology outpatient clinic between 2008 and 2012. All patients with age 15 years and above, of either gender and have been diagnosed as a case of ulcerative colitis were included. The diagnosis of UC was based on a combination of clinical, endoscopic, radiological and histopathological findings. As infective colitis can mimic the features of UC, this was regularly sought after and excluded. The baseline characteristics and extent of the disease were documented. Extent of colonic involvement was based on colonoscopy and biopsy findings and was classified as follows: proctitis (limited to the rectum), left-sided colitis (a disease that extends beyond the rectum and as far proximally as the splenic flexure), extensive colitis (disease involvement extending beyond the splenic flexure but not involving the cecum) and pancolitis (disease involving the entire colon). The Mayo scoring system was used to judge disease severity. It is composed of four categories (rectal bleeding, stool frequency, physician assessment, and endoscopic appearance) rated from 0 to 3 that are summed to give a total score that ranges from 0 to 12. The higher scores correlating with more severe disease.^6^ The disease activity was labeled as mild with a score of 3 to 5, moderate to severe when the score was 6 to 12. Values were expressed as mean ± SD for continuous variables and number with percentages for categorical variables. Data were analyzed using statistic of package for social sciences (SPSS 19) program (Chicago, IL, USA).

## RESULTS AND DISCUSSION

**Graph 1:**
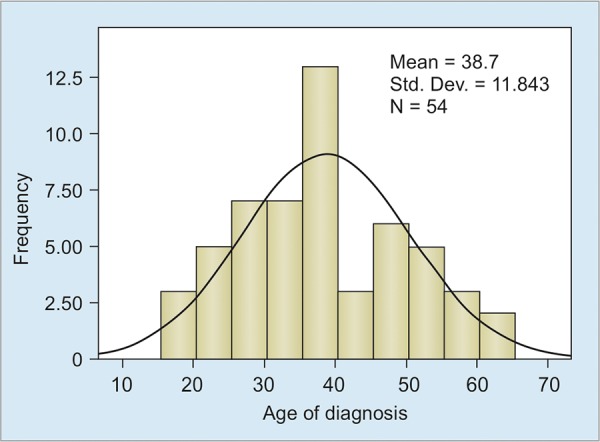
Age distribution of study patients

A total of 54 patients with UC were included in the study, of whom 27 were males and 27 were females; giving a male to female ratio of 1:1. Mean age of patients at the time of diagnosis of UC was 38.7 ± 11.8 years (median 36.5, range 18-64) and at the time of data analysis was 43.8 ± 12.8 years (median 42, range 18-75). The peak age of onset was the fourth decade (Graph 1). Only one patient had a positive family history (1.85%).

**Table 1:** **Table 1:** Clinical characteristics of study patients

Gender (M:F)		27:27	
Age in years: median (range)		36.5 (18-64)	
Mean ± SD		38.7 ± 11.8	
*Presenting symptom*		*%(n)*	
Mucous diarrhea		90.7 (49)	
Gross blood in stool		77.8 (42)	
Abdominal pain or cramps		74.1 (40)	
Weight loss		27.7 (15)	
Severe bowel movement urgency		3.7 (02)	
Vomiting		3.7 (02)	
*Extent of disease*			
Proctitis		9.2 (5)	
Left-sided colitis		42.6 (23)	
Extensive colitis		20.4 (11)	
Pancolitis		27.8 (15)	
*Disease severity*			
Mild		31.5 (17)	
Moderate to severe		68.5 (37)	

**Graph 2:**
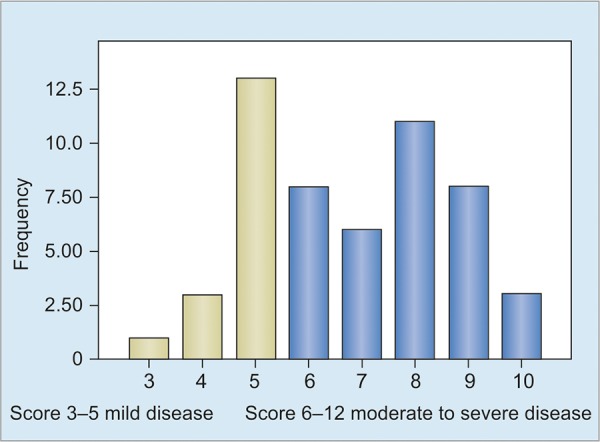
Disease severity according to mayo scoring system

The predominant presenting symptoms were mucus diarrhea in 90.7%, gross blood in stools in 77.8% and abdominal pain or cramps in 74.1% (Table 1). Other less frequent symptoms in UC included weight loss (22.7%), severe bowel movement urgency (3.7%) and vomiting (3.7%). The extraintestinal manifestations were found only in seven patients; arthritis in five and anterior uveitis in two patients. The arthritis was unilateral and the sites were knee joint in three patients and sacroiliac joint in two patients. None of the patients had sclerosing cholangitis or colorectal cancer. Extent of colonic involvement was based on colonoscopy and biopsy findings. The extent of the disease was proctitis in 9.2%, left-sided colitis in 42.6%, extensive colitis in 20.4% and pancolitis in 27.8%. The severity of UC was judged by the Mayo scoring system with a score range 0 to 12, the higher scores correlating with more severe disease. Our study revealed that 68.5% were categorized in moderate to severe disease (score 6-12) and 31.48% had mild disease (score 3-5) (Graph 2).

Conventionally, IBD has been considered to be a rare disease in the Asia-Pacific region, but recent evidence indicates that both Crohn’s disease and ulcerative colitis are becoming increasingly common in Asian populations.^7^ In our study, the distribution of UC is equal in males and females. Studies from China, Japan and Korea have confirmed our observation that the gender distribution for UC is equal among Asians.^8,9^ Mean age at the time of diagnosis of UC was 38.5 years. This is similar to other Asian countries like South Korea (35 years),^7^ Thailand (33 years)^10^ and China (44 years).^11^

In our study, the predominant chief complaints were mucous diarrhea, gross blood in the stool and abdominal pain. There was no difference in the clinical manifestations of the colonic disease in our patients from other Asian or developed countries.^12^ Most studies have shown that UC patients have less extracolonic manifestations in Asian countries. Pokharna et al prospectively studied extraintestinal manifestations of UC in Indian patients from Rajasthan.^13^ Out of 46 patients, one patient (2%) had peripheral arthritis and two patients (4%) had ocular involvement in the form of anterior uveitis. Similarly, a low frequency of extraintestinal manifestations has been reported from China (6.1°%) and Singapore.^14^ In our study the extraintestinal manifestations were 12.9% and more common were arthritis and anterior uveitis. None of the patients in this series had sclerosing chol-angitis and colorectal cancer. This rate of extraintestinal manifestations is much lower than 40 to 50% reported in studies from the developed countries.^15^ There was only one patient who had a positive family history of disease, suggesting reduced familial susceptibility.

Left-sided colitis was the most common presentation in our study and in other studies from China (70.2%), India (47.5%) and Thailand (58%).^16-18^ In contrast, pan-colitis was more common in patients from Kuwait (45%) and Singapore (37.6%).^19,20^ Therefore, there is no clear-cut confirmation that the extent of UC is different among Asian and Caucasian populations. In our study, 68.5% cases could be categorized as having moderate to severe disease based on the Mayo scoring system. This matches with a study from India where the majority of the patients had severe clinical, endoscopic and histologic disease commensurate with other recent studies.^21^ In contrast, milder presentation was seen in some earlier studies from Kuwait and China.^14,19^

In a study from Pakistan published 23 years back, the majority of patients were in the 21 to 30 years age group, the age at the time of diagnosis was younger than our patients. There was no gender preponderance like our study. Seventy-two percent had mild to moderate disease in contrast to our study where the majority had moderate to severe disease. The disease was mainly confined to the left colon in 60 and 13% had total colitis.^22^ The extents of disease was in consistence with our study. More severe presentation in our study as compared to previous one may be related to changes in lifestyle and dietary habits over the years. A similar shift has been reported in another paper from our neighboring country.^21^ However, further studies are necessary for better understanding of the clinical course of UC in Pakistan.

## CONCLUSION

Ulcerative colitis presents in our adult patients may present at any age, mostly young and middle age patients with no gender preponderance. The disease severity is moderate to severe in the majority of patients and more than half of them have left-sided colitis or pancolitis at the time of presentation. Extraintestinal manifestations were not common.
